# Compare the Effect of Inhaled Corticosteroids and Systemic Corticosteroids on Sputum Microbiome of AECOPD

**DOI:** 10.3389/fmed.2021.637246

**Published:** 2021-02-26

**Authors:** Nan Ma, Yujing Qi, Xiaona Liang, Jing Bai, Jingmin Deng, Meihua Li, Zhiyi He

**Affiliations:** Department of Pulmonary and Critical Care Medicine, First Affiliated Hospital of Guangxi Medical University, Nanning, China

**Keywords:** chronic obstructive pulmonary disease, acute exacerbation, inhaled corticosteroids, systemic corticosteroids, sputum microbiome

## Abstract

**Background:** To observe the effects of inhaled corticosteroids (ICS) and systemic corticosteroids (SCS) on the sputum microbiology of patients with AECOPD.

**Methods:** The 16S rRNA sequencing results for sputum samples from 36 admitted AECOPD patients were analyzed using ICS or SCS on the basis of standard treatment; sputum samples were collected before and after treatment for 1 day, 7, and 14 days.

**Results:** After 7 days of SCS treatment, the bacterial abundance of *Sorangium, Acidibacter*, and *Fretibacterium* decreased at the genus level. After 14 days of SCS treatment, the bacterial abundance of *Prevotella_2, Bergeyella, Corynebacterium_1*, and *Ruminococcaceae_UCG-014* was decreased at the genus level, and an increase in the bacterial abundance of the Clostridiales_vadinBB60_group was observed at the family level. The linear discriminant analysis effect size (LEfSe) algorithm showed that after treatment for 14 days, *Sphingobacterium* increased in the SCS group, and Corynebacterium_1 (genus level), Bacillales (order level), and Lactobacillales (order level) decreased in the ICS group. However, the abundance of the above bacteria in each group of samples was <1%, suggesting that the two treatments may have similar effects on bacterial abundance. Alpha diversity analysis results showed that there was no significant difference in the ACE index, Chao1 index, Shannon index, or Simpson index between the ICS group and the SCS group. Beta diversity analysis showed that there was little difference in bacterial diversity among each group. BugBase predicted that although bacteria containing mobile elements in the SCS group decreased significantly compared with those in patients using ICS after treatment for 14 days, these two treatments had similar effects on other phenotype categories assigned to the bacterial contents.

**Conclusions:** Our results show that ICS and SCS have remarkably similar effects on the sputum microbiome of AECOPD patients.

## Introduction

Chronic obstructive pulmonary disease (COPD) is a common respiratory disease that is characterized by persistent symptoms and impaired lung function due to small airway obliteration and alveolar destruction and is related to a chronic inflammatory response in the lungs (1, 2). The exacerbation of airway inflammation and disease progression during acute exacerbation of COPD have been demonstrated to be related to changes in the lung microbiology (3). The effects of acute exacerbation of COPD on airway microbiology mainly consist of the imbalance of airway microbiology and changes in microecological diversity (1, 3, 4).

Glucocorticoids are widely used to treat a variety of pulmonary inflammatory diseases and have become first-line therapies for COPD (5). An enhanced systemic inflammatory response is a pathological characteristic of AECOPD, and glucocorticoids show anti-inflammatory effects in patients with AECOPD. Systemic glucocorticoids (SCS) are recommended by guidelines for the treatment of AECOPD (5, 6), and SCS improve oxygenation, the risk of early relapse, treatment failure, and the length of hospitalization (5, 7, 8). The Global Initiative for Chronic Obstructive Lung Disease notes that therapy with inhaled corticosteroids (ICS) is equally as effective as SCS during the acute exacerbation of COPD (9). The current study demonstrated that 6–8 mg of corticosteroids administered by nebulized inhalation provides benefits and short-term clinical outcomes similar to those of systemic corticosteroids (10). In the treatment of AECOPD, the advantage of ICS lies in the lower incidence of adverse events than SCS (11), such as impairment of systemic immunity, infection, steroid diabetes, and osteoporosis (10).

Corticosteroid treatment of AECOPD has significant effects on microbiome composition, and treatment with SCS alone and without antibiotics can increase Proteobacteria, Bacteroides, and Firmicute abundance (12). One-year treatment with ICS increases the total bacterial load in the sputum of COPD patients and changes the microbiome composition (13). Local inflammation in AECOPD is related to changes in the microbiota, characterized by reduced microbial diversity (14, 15). Treatment with SCS alone decreased microbial alpha diversity in AECOPD (1). Long-term use of ICS in COPD patients is positively correlated with beta diversity of the upper airway microbiota (16). However, whether there are different effects of ICS and SCS on the sputum microbiome during the treatment of AECOPD remains poorly understood. This study investigated the bacterial load and microbial diversity and aimed to compare the effects of ICS and SCS on the sputum microbiome in patients with AECOPD.

## Materials and Methods

### Ethical Approval

This study was approved by the Ethics Committee of the First Affiliated Hospital of Guangxi Medical University in 2017 (number: 2017KY-E-024). All subjects provided written informed consent before enrollment, and all subjects could withdraw from the study at any time.

### Patients

Thirty-six patients with AECOPD meeting the diagnostic criteria of GOLD (17) were enrolled in the present study. There are no antibiotic treatment bofore patients admitted to hospital. The inclusion criteria of patients were as follows: (1) patients were more than 50 years old and had a history of smoking or smoking exposure; (2) patients were diagnosed with acute exacerbation of COPD that requires additional treatment; (3) patients had no history of asthma, tuberculosis, cystic fibrosis, obliterative bronchiolitis, pulmonary embolism, diffuse panbronchiolitis (DPB), and no complications with acute respiratory failure, acidosis, cancer, or serious heart, liver, or kidney disease; and (4) patients were able to undergo pulmonary function tests or venipuncture.

### Study Design

Patients with AECOPD were randomly assigned to the ICS group and SCS group, patients in the ICS group received inhaled budesonide (2 mg Tid for 5 days), and patients in the SCS group were treated with systemic methylprednisolone (methylprednisolone acetate injectable suspension 40 mg Qd for 5 days). Therapy with corticosteroids was based on standard treatments, including long-acting muscarinic antagonists (LAMAs), long-acting beta-adrenergic receptor agonists (LABA), broad-spectrum antibiotics, and low-flow oxygen, according to local treatment guidelines for AECOPD (17).

### Sputum Sampling and Microbiome Analysis

As per the inclusion criteria, sputum samples were collected for all patients on the 1st, 7th, and 14th mornings before glucocorticoid use and ICS/SCS treatment. To collect induced sputum, 3% sterile NaCl solution was inhaled by nebulizer for 15 min, and taked the induced sputum, it was the same as those introduced in our previous paper (18). And bacterial 16S rRNA gene detection were also the same as our previous paper (18).

### Bioinformatic and Statistical Analysis

Usearch software (v.10.0.240) was used to identify unique tags and to discard excess tags, and tags with more than 97% similarity were clustered into operational taxonomic units (OTUs) by Usearch (19–21). Krona taxonomy visualization and petals diagrams were used to analyze the relative proportions of different OTUs for different classification levels (22). Metastats software was used to analyze bacteria with different abundances at the family and genus levels (23). Linear discriminant analysis effect size (LEfSe) was used to determine which OTUs had differences between the groups under different biological conditions. LEfSe is suitable for the discovery and interpretation of multilevel biological identification and characteristics, such as species taxonomy pedigrees (24). The Shannon sparse curve was drawn using the Shannon index, which is used to evaluate whether the amount of sequencing data is sufficient to cover all bacterial species and reflect the species richness in the sample (25, 26). Alpha diversity was calculated on the basis of the gene profile for each sample based on the Shannon, Chao1, Simpson, and ACE indices. Alpha diversity estimates were computed using mothur software (version 1.30) and R (version 3.5.1), beta diversity estimates were computed using QIIME1 (v1.8.0) software, and non-metric multidimensional scaling (NMDS) based on Bray-Curtis dissimilarity was used to visualize potential clustering patterns among samples based on the estimated beta diversity and its relationship with dominant taxa. BugBase was used to predict bacterial composition based on the sequencing results.

To determine the differentially expressed OTUs between samples, the R package pheatmap was used to construct a heatmap for the OTU expression profile. Spearman rank correlation analysis was used to screen the bacteria demonstrating correlations (family level, HR > 0.1 and *p* < 0.05), and the microbial network was constructed by Python.

## Results

### Patient Characteristics

Eighteen ICS patients and 18 SCS patients were enrolled and eligible for this research study. There were no significant differences in basic clinical characteristics between the two groups (age, smoking status, BMI, lung function, CAT, WBC, NEU, Sex, *P* > 0.05), as shown in Table 1.

**Table 1:** Major clinical characteristics of patients in the cohort upon admission.

	**ICS group (*n* = 18)**	**SCS group (*n* = 18)**	***p*-value**
**Age (year)**	67.72±6.95	66.47±7.4	0.609
**Smoke (pack-years)**	90.44±85.02	62.29±21.67	0.195
**BMI (kg/m^2^)**	22.17±2.36	21.70±2.70	0.589
**FEV1%pred**	39.17±17.48	37.70±16.29	0.799
**FEV1/FVC**	44.29±16.44	43.14±13.92	0.824
**CAT**	20.17±4.32	18.47±3.78	0.226
**WBC**	8.87±1.72	8.96±1.93	0.875
**NEU**	6.58±1.26	6.78±1.07	0.619
**Gender**			0.471
Male	14	11	
Female	4	7	
**GOLD**			
D	9	9	
C	5	5	
B	4	3	
**mMRC**			
3	2	4	
2	8	8	
1	8	5	

### Comparison of the Effects of ICS and SCS on Lung Bacterial Load in Patients With AECOPD

**Figure 1:**
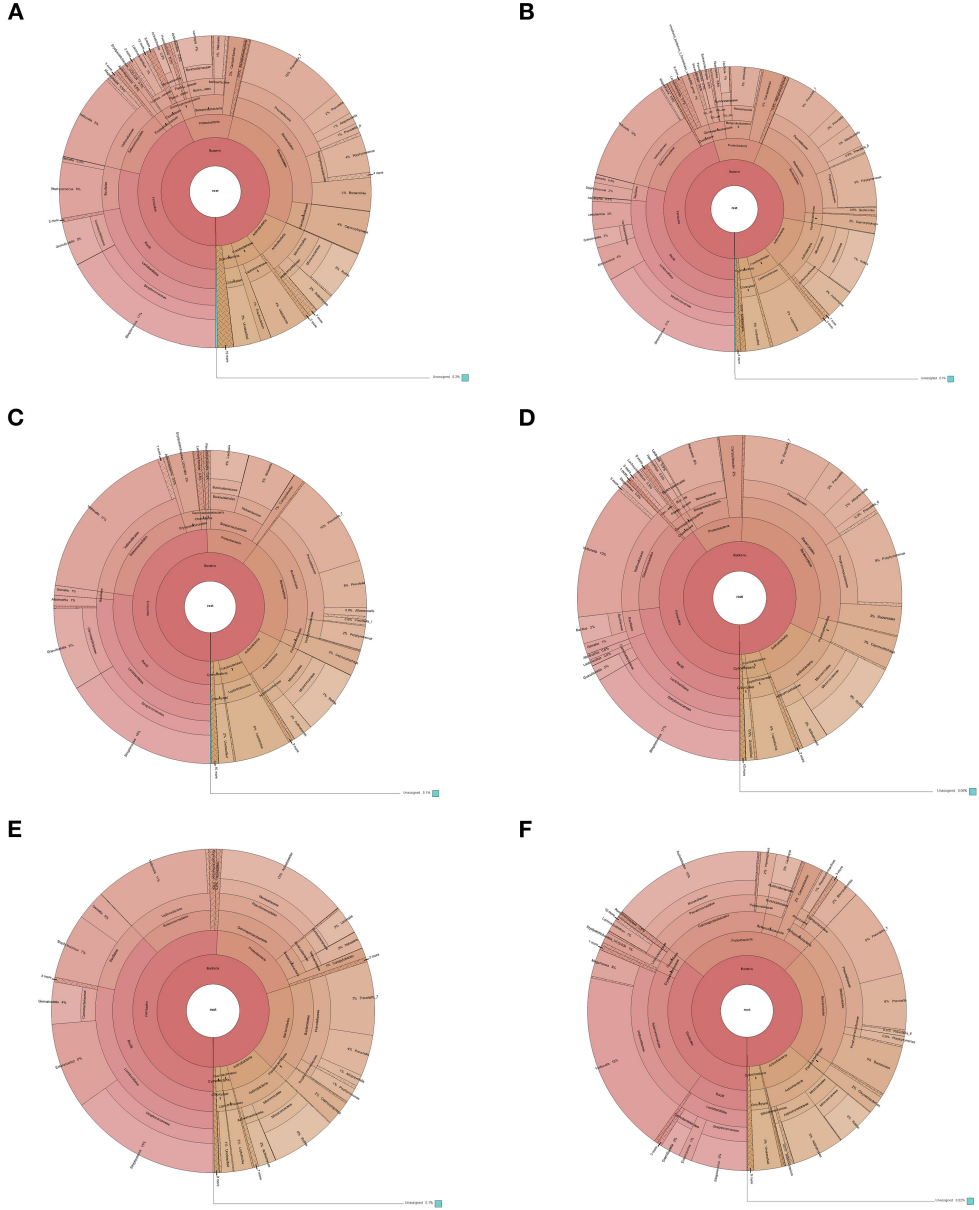
Species distribution of each group. Krona is taxonomy web visualization; circles from inside to outside represent different classification levels, and the area of the sector indicates the proportion of different OTU annotation results. Rings from inside to outside: Contents of various bacteria at the kingdom, phylum, class, order, family, and genus levels. **(A)**: ICS 1 day. **(B)**: SCS 1 day. **(C)**: ICS 7 day. **(D)**: SCS 7 day. **(E)**: ICS 14 day. **(F)**: SCS 14 day.

**Figure 2:**
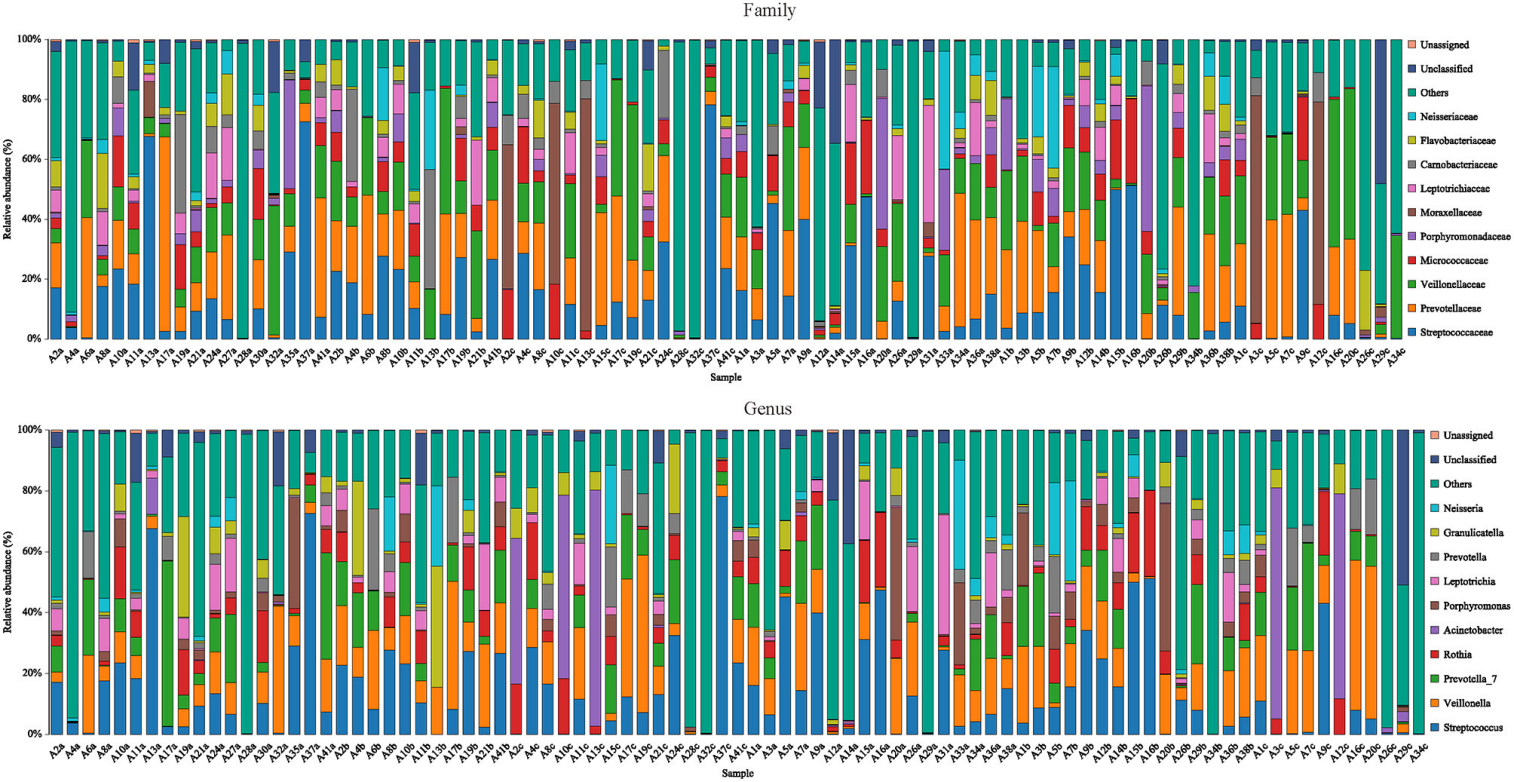
Species distribution of each sample. Species distribution of bacteria in each sample (family and genus level). Community composition based on 16S rRNA gene fragment sequences in sputum samples. Bars represent the relative abundance of the most abundant families (>5% in one sample). The remaining families are subsumed as others.

**Figure 3:**
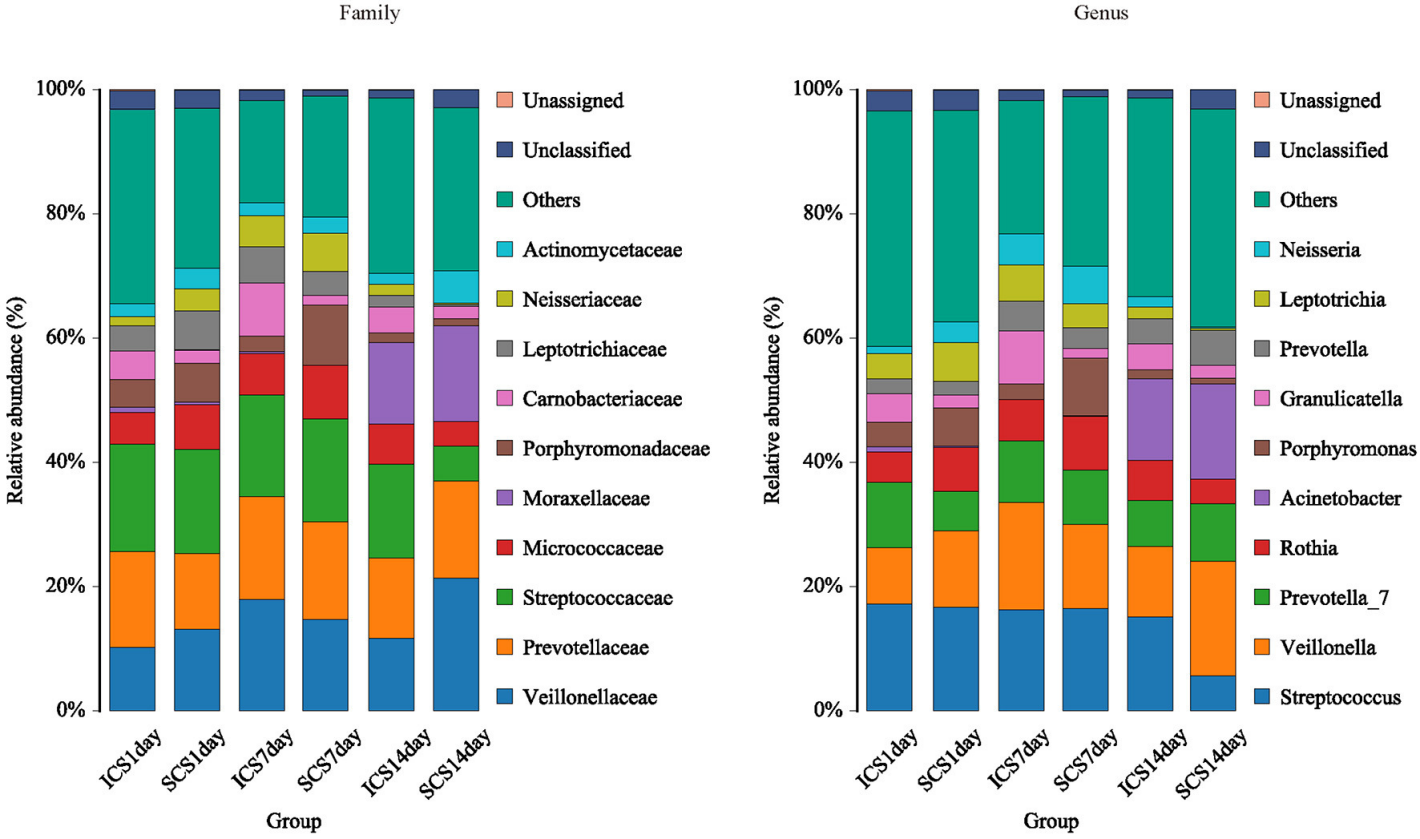
Species distribution of each group. Species distribution of bacteria in each group (family and genus level). Community composition based on 16S rRNA gene fragment sequences in sputum samples. Bars represent the relative abundances of the most abundant families (>5% in one group). The remaining families are subsumed as others.

**Figure 4:**
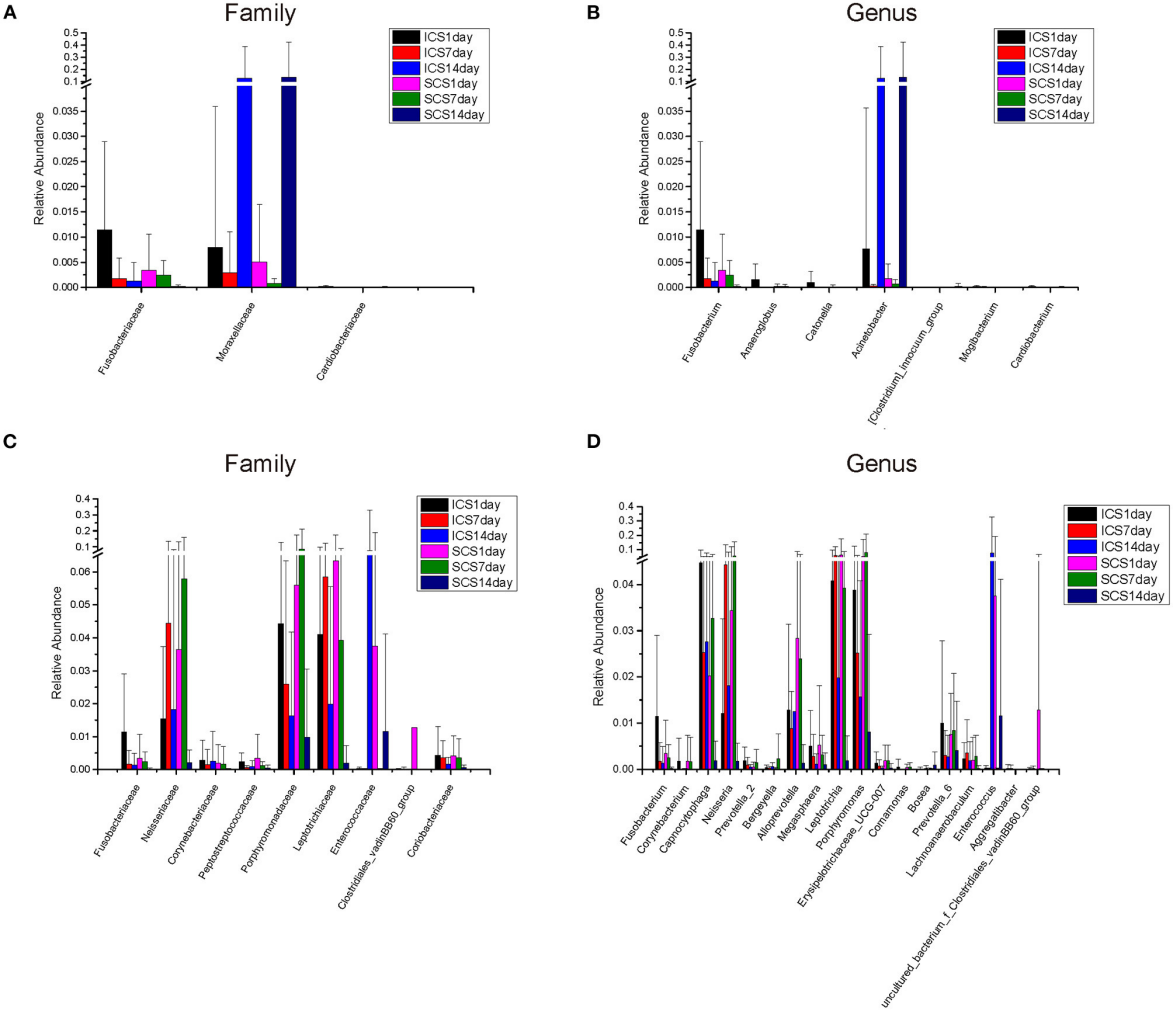
Colonies with different abundances. **(A,B)** Colonies with different abundances in each group. Statistical analyses were calculated by one-way analysis of variance by ANOVA (family and genus levels, *p* < 0.05). **(C,D)** Colonies with different abundances in each group. Statistical analyses were calculated by the Wilcoxon test (family and genus levels, *p* < 0.05).

**Figure 5:**
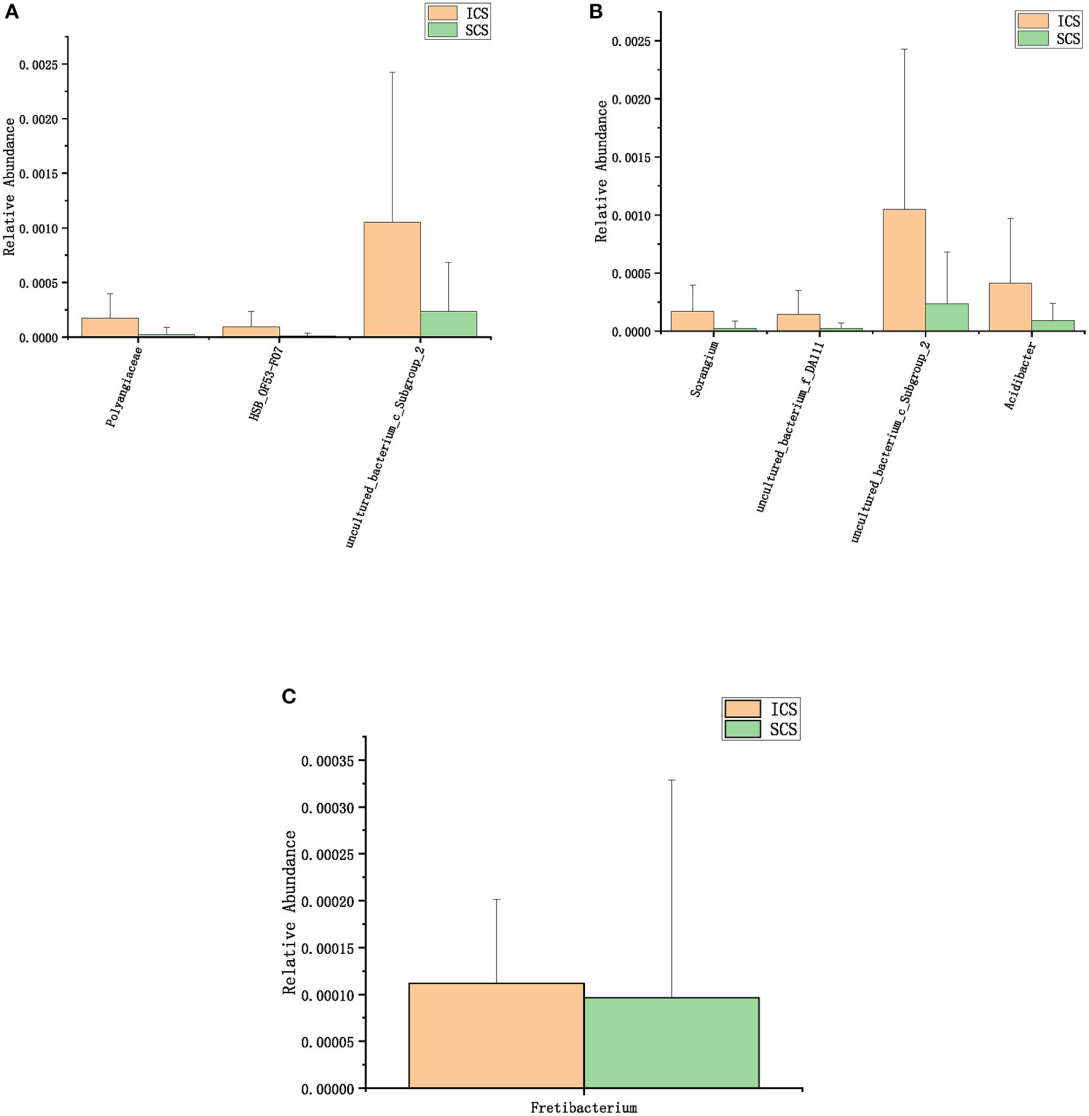
Colonies with different abundances after 7 days of treatment. **(A)** One-way analysis of variance (ANOVA) at the family level. **(B)** Calculated by ANOVA at the genus level. **(C)** Calculated by Wilcoxon test at the genus level (*p* < 0.05).

**Figure 6:**
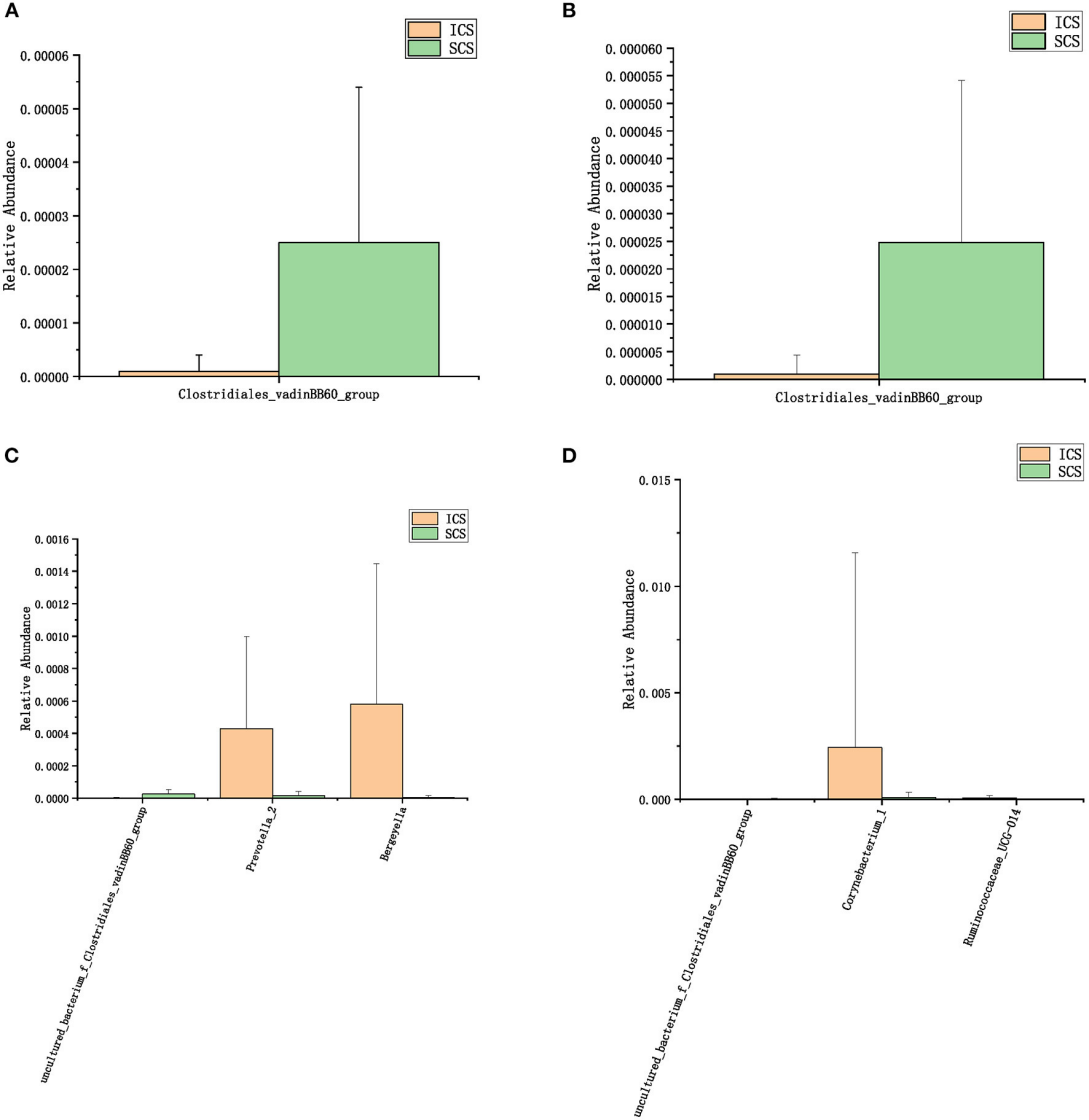
Colonies with different abundances after 14 days of treatment. **(A)**: One-way analysis of variance by ANOVA at the family level. **(B)**: Calculated by Wilcoxon test at the family level. **(C)**: One-way analysis of variance (ANOVA) at the genus level. **(D)**: Calculated by Wilcoxon test at the genus level. (*p* < 0.05).

The species annotation of each group was visualized by using KRONA (Figure 1), and a histogram was generated to show the species distribution of each sample (Figure 2) and each group (Figure 3). ANOVA and Wilcoxon tests were used to analyze the effects of ICS and SCS on the lung bacterial load of patients with COPD. An ANOVA test showed that the colonies with different abundances at the family level were *Fusobacteriaceae, Moraxellaceae*, and *Cardiobacteriaceae*, while *Fusobacterium, Anaeroglobus, Catonella, Acinetobacter, [Clostridium]_innocuum_group, Mogibacterium*, and *Cardiobacterium* showed different abundances at the genus level; the Wilcoxon test showed that colonies with different abundances at the family level were *Fusobacteriaceae, Neisseriaceae, Corynebacteriaceae, Peptostreptococcaceae, Porphyromonadaceae, Leptotrichiaceae, Enterococcaceae, Clostridiales_vadinBB60_group*, and *Coriobacteriaceae*, while *Fusobacterium, Corynebacterium, Capnocytophaga, Neisseria, Prevotella_2, Bergeyella, Alloprevotella, Megasphaera, Leptotrichia, Porphyromonas, Erysipelotrichaceae_UCG-007, Comamonas, Bosea, Prevotella_6, Lachnoanaerobaculum, Enterococcus, Aggregatibacter*, and *uncultured_bacterium_f_Clostridiales_vadinBB60_group* showed different abundances at the genus level (Figure 4, *p* < 0.05). After 7 days of SCS treatment, the bacterial abundances of *Sorangium, Acidibacter*, and *Fretibacterium* decreased at the genus level (Figure 5, *p* < 0.05). After 14 days of SCS treatment, the bacterial abundances of *Prevotella_2, Bergeyella, Corynebacterium_1*, and *Ruminococcaceae_UCG-014* decreased at the genus level, and an increase in the bacterial abundance of Clostridiales_vadinBB60_group was observed at the family level (Figure 6, *p* < 0.05).

**Figure 7:**
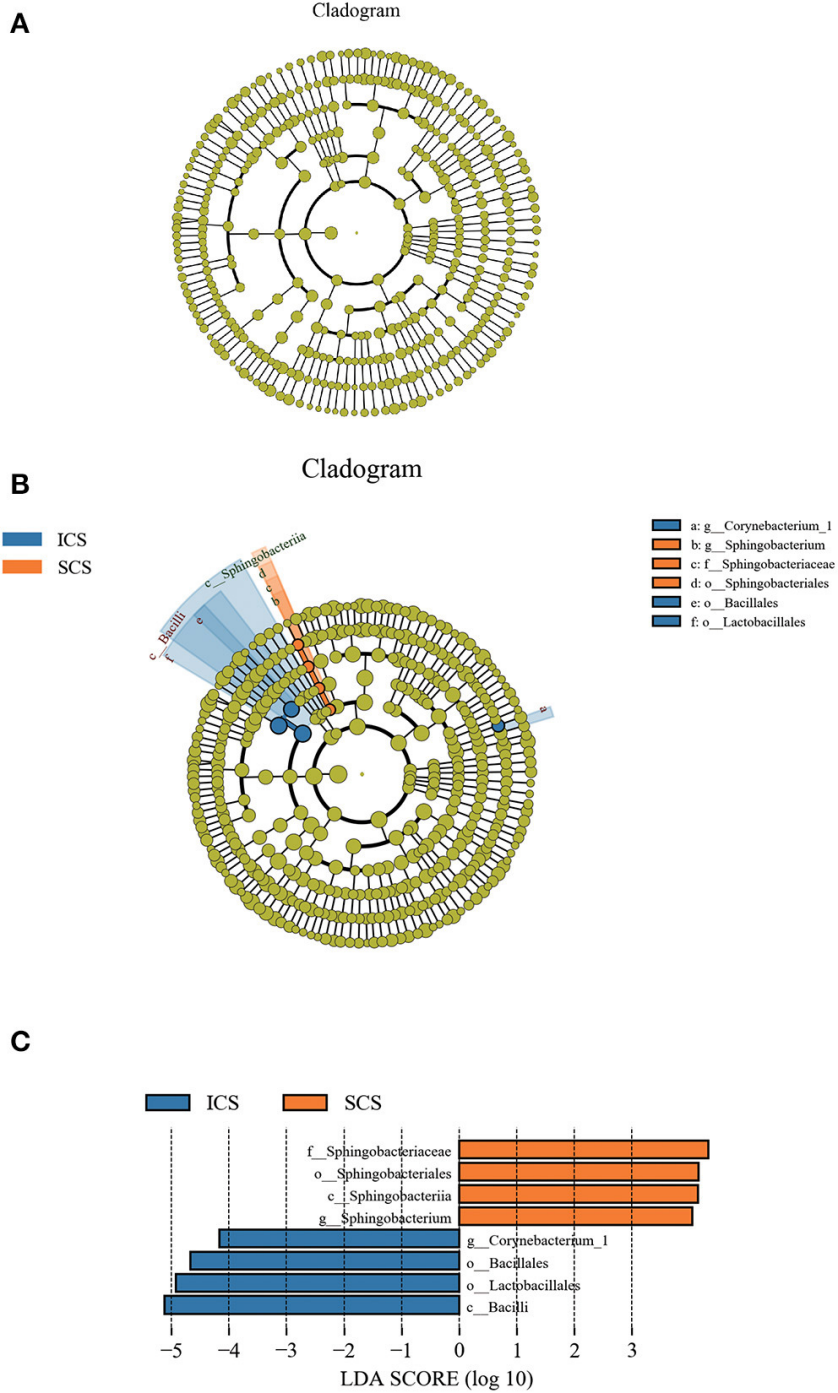
Differential bacterial abundance analyzed by linear discriminant analysis effect size (LEfSe). **(A)** Colonies with different abundances after treatment for 7 days. Our results showed no difference in abundance between the two groups. **(B,C)** Colonies with different abundances after treatment for 14 days, *Sphingobacterium* increased after treatment with SCS, and abundances of Corynebacterium_1 (genus level), Bacillales (order level), and Lactobacillales (order level) were decreased by ICS treatment (*p* < 0.05).

Differential taxonomic features between the ICS and SCS were calculated by linear discriminant analysis effect size (LefSe), and the results showed no difference in abundance between the two groups after treatment for 7 days (Figure 7A, *p* < 0.05). After treatment for 14 days, *Sphingobacterium* increased after treatment with SCS, and Corynebacterium_1 (genus level), Bacillales (order level), and Lactobacillales (order level) decreased following treatment with ICS (Figures 7B,C; *p* < 0.05). However, the content of bacteria with different abundances between the two groups was <1% in each group (Figure 1).

### The Influence of ICS and SCS on Lung Microecological Diversity in Patients With AECOPD

**Figure 8:**
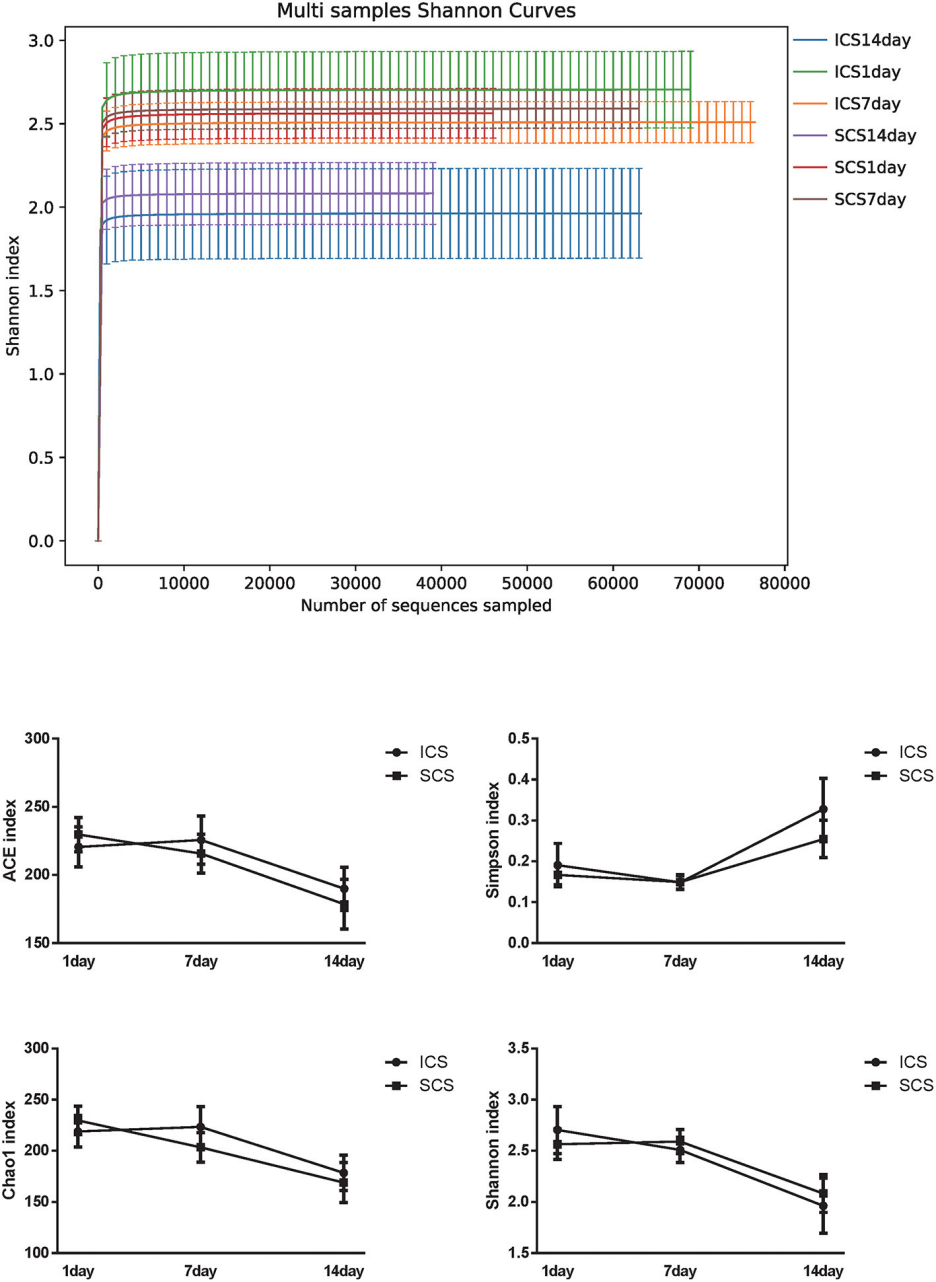
Alpha diversity in ICS vs. SCS. Shannon index of each group and statistical analysis of the ACE index, Chao1 index, Shannon index, and Simpson index (*p* < 0.05). After 14 days of treatment, the ACE index of the SCS group was significantly lower than the first day of treatment, and the Shannon index of the SCS group was significantly lower than the first day of treatment. The Shannon index of the ICS group was also significantly lower than the first day of treatment. No differences were found between the two groups at the same treatment time (*p* < 0.05).

Alpha diversity analysis results showed that after 14 days of treatment, the ACE index of the SCS group was significantly lower than the first day of treatment, and the Shannon index of the SCS group was significantly lower than the 7th day after treatment. It was also observed that the Shannon index of the ICS group was significantly lower than the first day of treatment. No differences were found between the two groups at the same treatment time (Figure 8, *p* < 0.05).

**Figure 9:**
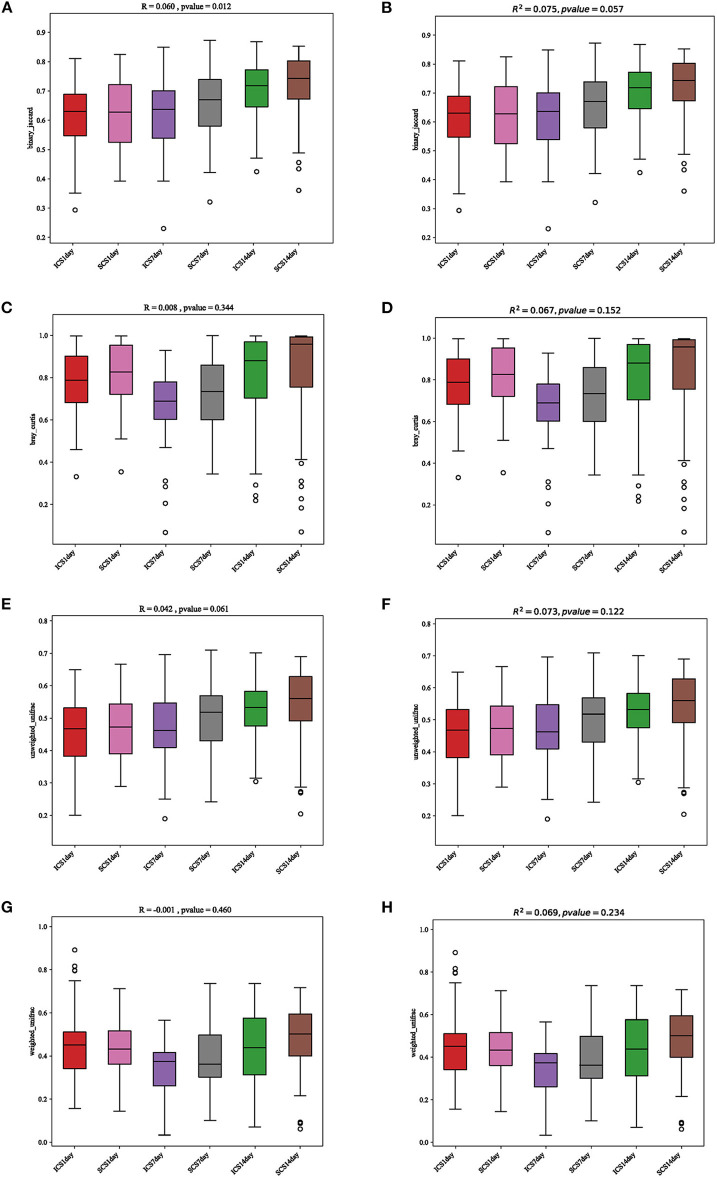
Statistical analysis results for PCA and PcoA. **(A)** Anosim statistical analysis of the results, analyzed by the binary Jaccard algorithm. **(B)** PERMANOVA statistical analysis of the results, analyzed by the binary Jaccard algorithm. **(C)** Anosim statistical analysis of the results, analyzed by the Bray-Curtis algorithm. **(D)** PERMANOVA statistical analysis of the results, analyzed by the Bray-Curtis algorithm. **(E)** Anosim statistical analysis of the results, analyzed by the unweighted UniFrac algorithm. **(F)** PERMANOVA statistical analysis of the results, analyzed by the unweighted UniFrac algorithm. **(G)** Anosim statistical analysis of the results, analyzed by the weighted UniFrac algorithm. **(H)** PERMANOVA statistical analysis of the results, analyzed by the weighted UniFrac algorithm. The results showed no differences between the two groups, and there was no significant difference before or after treatment.

**Figure 10:**
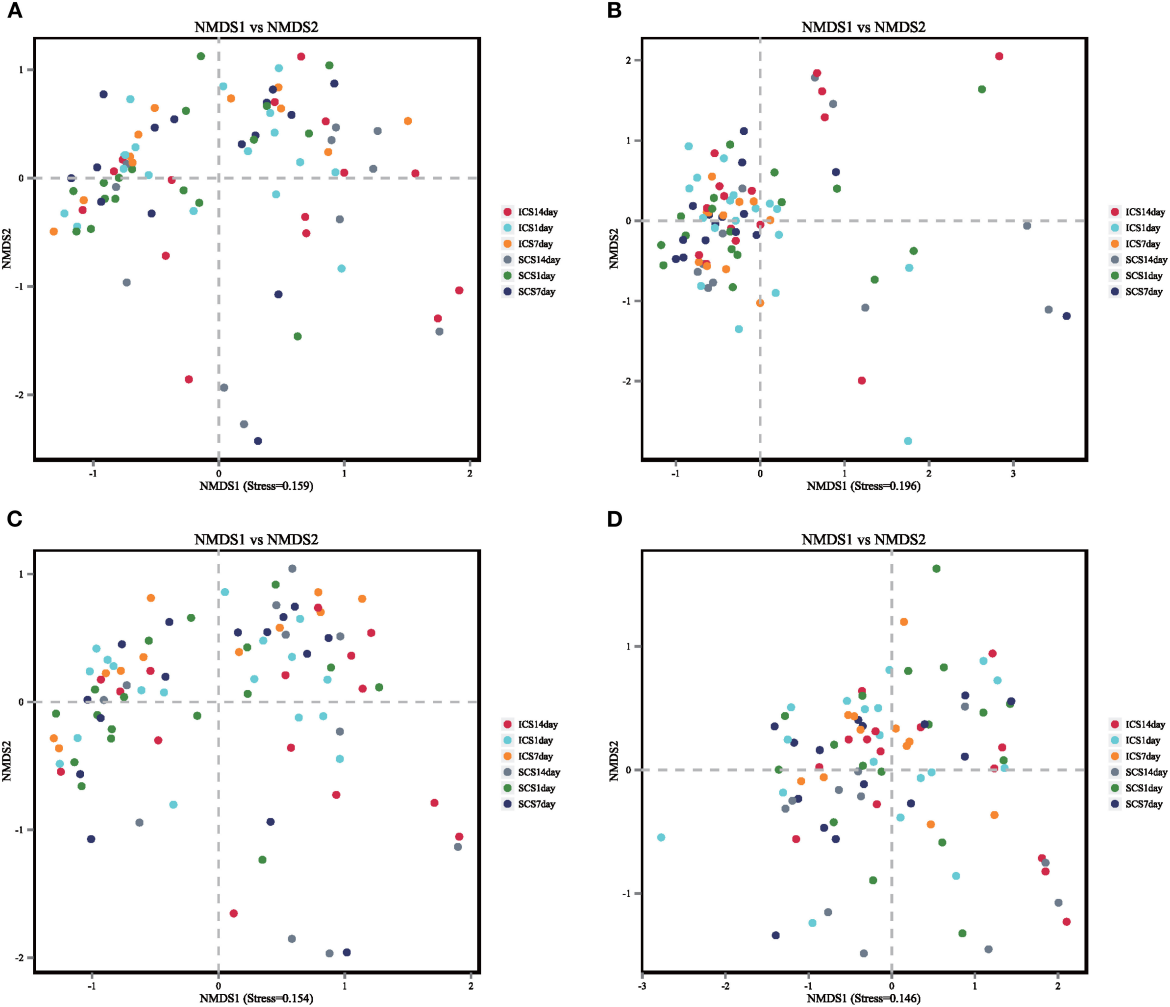
Non-metric multidimensional scaling (NMDS) was used to analyze the colony diversity with the ICS and SCS treatments. The results of NMDS showed that there was no significant difference in bacterial diversity among groups, and ICS and SCS had the same effect on colony diversity via NMDS. Stress < 0.2 in this figure, suggesting that the NMDS analysis is reliable.

In the beta diversity analysis, PCA, PcoA, and nonmetric multidimensional scaling (NMDS) analyze of bacteria showed no significant divisions into groups (Figure 9); Anosim and PERMANOVA statistical analyses showed there was no significant difference in bacterial diversity among the groups and therefore, no significant difference in beta diversity between the two groups (Figure 10, *p* < 0.05).

### The Influence of ICS and SCS on Microbiome Characteristics

**Figure 11:**
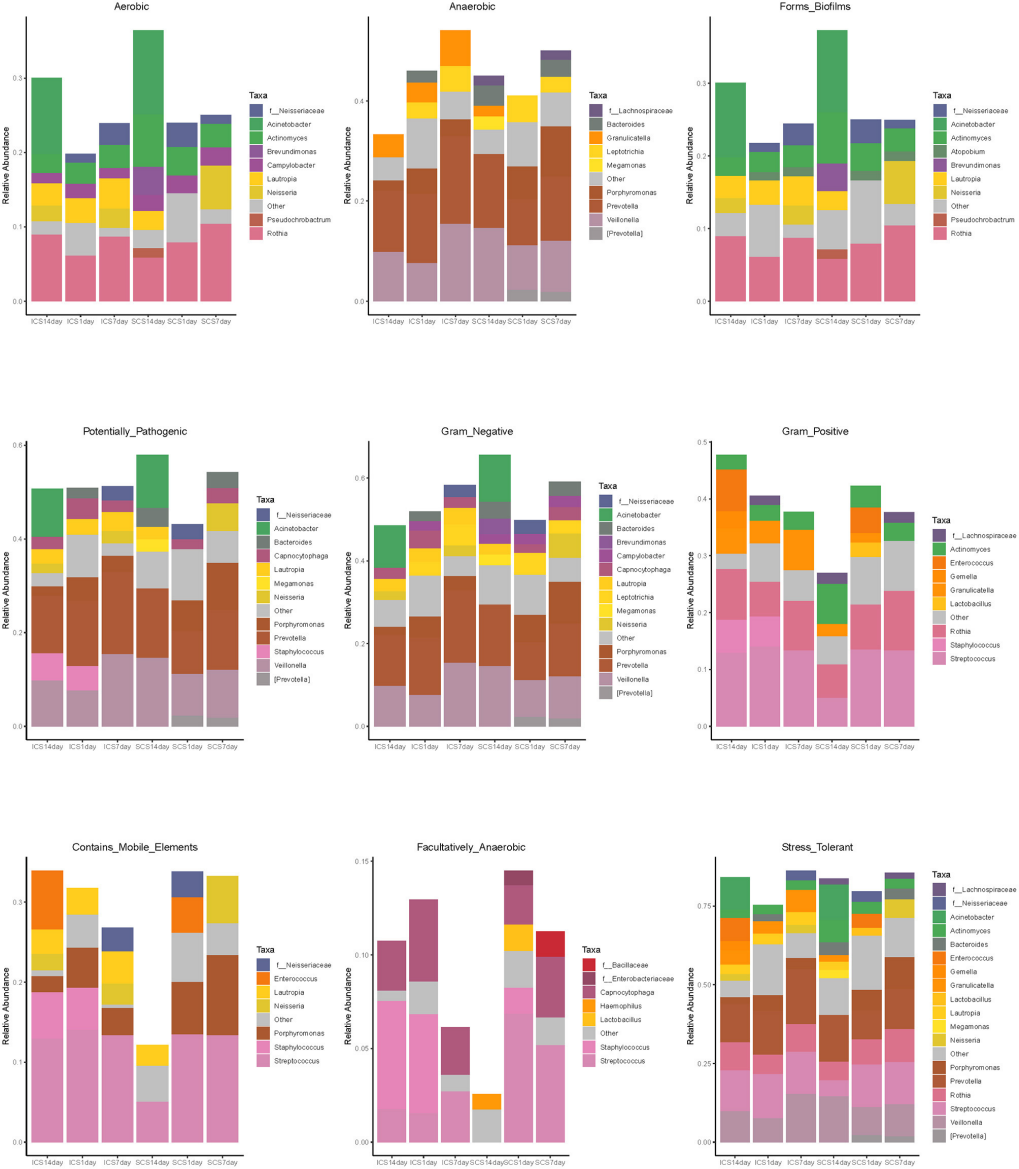
Bacterial composition of each group predicted by BugBase. Bacterial composition of each group, including Gram Positive, Gram Negative, Biofilm Forming, Pathogenic Potential, Mobile Element Containing, Oxygen Utilizing, and Oxidative Stress Tolerant.

**Figure 12:**
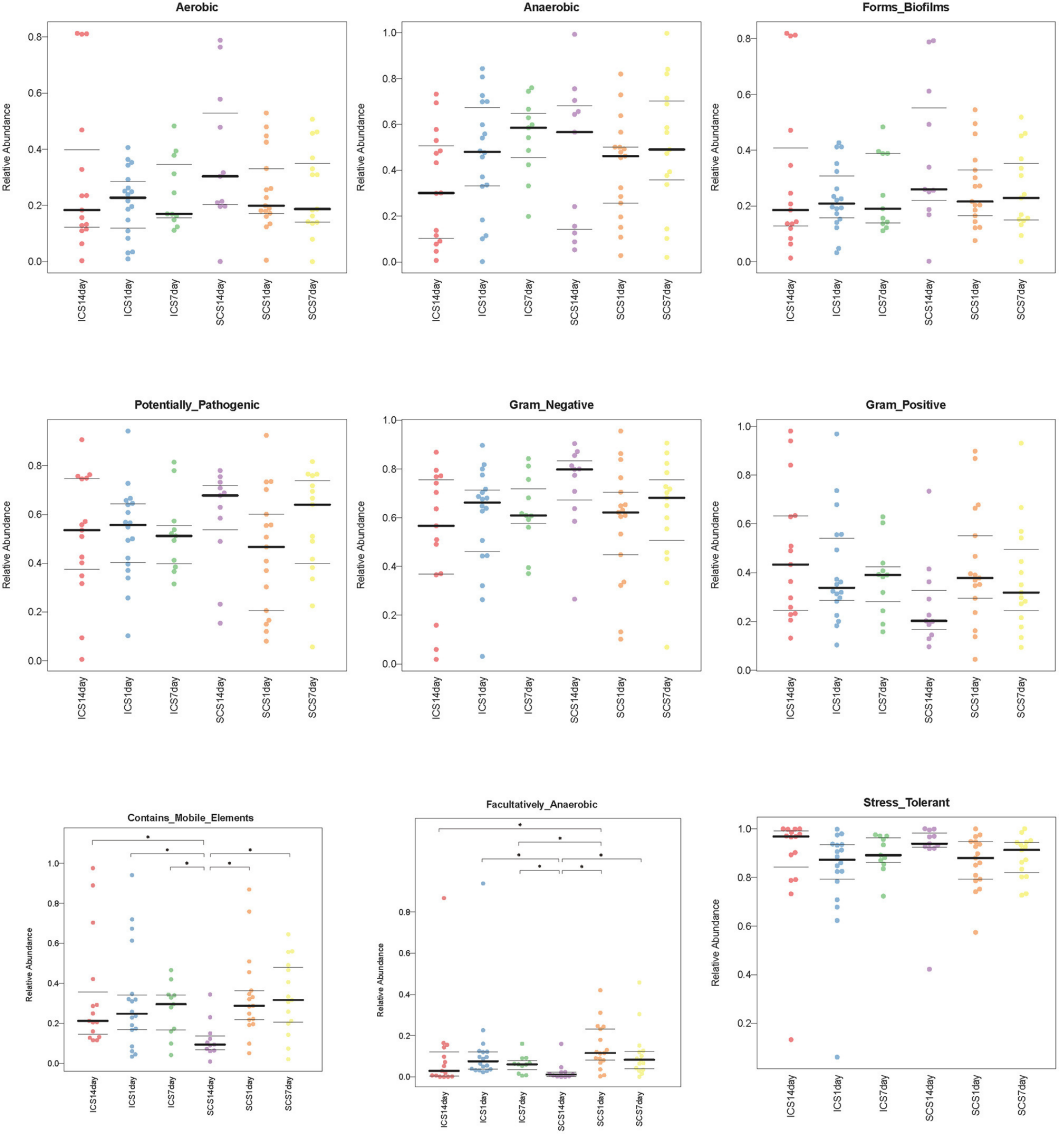
Compared the bacterial composition predicted by BugBase in two groups. Significant differences can be found in facultatively anaerobic content and mobile elements (*p* < 0.05). After treatment for 14 days, the levels of mobile element genes were decreased in the SCS group compared with the ICS group. ICS and SCS have the same effect on aerobes, anaerobes, potentially pathogenic bacteria, bacteria related to biofilm formation, gram-negative bacteria, gram-positive bacteria, facultative anaerobes, and oxidative stress-tolerant bacteria. The data were summarized as the quartile and mean.

BugBase predicted the bacterial composition of each group (Figure 11). After 14 days of treatment, the mobile genetic element (MGE) content in sputum samples of SCS patients was decreased significantly compared with that in patients using ICS. ICS and SCS had the same effects on aerobes, anaerobes, potentially pathogenic bacteria, bacteria related to biofilm formation, gram-negative bacteria, gram-positive bacteria, facultative anaerobes, and oxidative stress-tolerant bacteria (Figure 12; *p* < 0.05).

### Network Analyses Reveal Potential Microbiota Interactions

**Figure 13:**
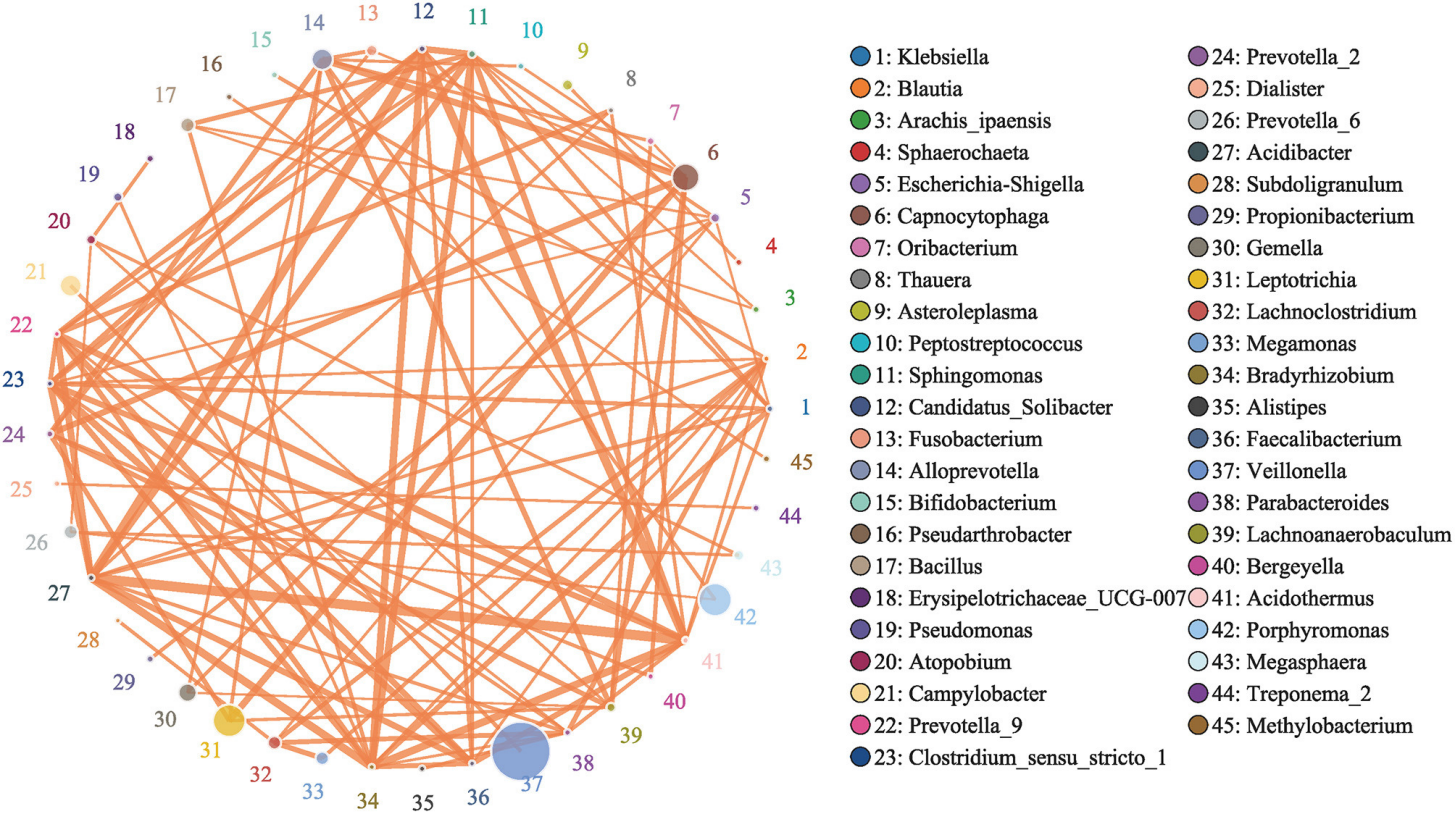
Network of Bacteria. Analyzed by Spearman rank correlation analysis. The size of the circle represents the average abundance of the species; the line represents the correlation between the two species; the thickness of the line represents the strength of the correlation; and regarding the color of the line, orange represents a positive correlation.

To study the interactions between bacteria, a bacterial network was constructed. The size of the circle represents the average abundance of the species; the line between circles represents the correlation between the two species, and the thicker the line, the stronger the correlation; finally, regarding the color of the line, orange represents a positive correlation, and green represents a negative correlation (Figure 13).

## Discussion

In this study, we observed the effects of corticosteroids on the pulmonary microbiology in patients with AECOPD. Corticosteroids are the main anti-inflammatory drugs for COPD, although most COPD patients have a poor response to corticosteroids (27, 28). Increasing evidence has demonstrated that high-dose ICS use in COPD patients increases the risk of pneumonia (29, 30). Studies have shown that the longer the course of SCS in AECOPD, the higher the risk of pneumonia (31). These studies suggest that glucocorticoids have an impact on lung microbiology in the treatment of COPD. However, the effect of ICS on airway microbiology in acute exacerbation of COPD remains unclear. To our knowledge, our research is the first to study the difference in the efficacy of ICS and SCS on the sputum microbiome of AECOPD patients.

We analyzed the bacterial 16S rRNA *via* bioinformatics methods, and the results showed that the frequently amplified families in the induced sputum of these patients were Streptococcaceae, Veillonellaceae, Prevotellaceae, Micrococcaceae, Moraxellaceae, Porphyromonadaceae, Carnobacteriaceae, Neisseriaceae, Leptotrichia, and Actinomycetaceae; at the genus level, they were *Streptococcus, Veillonella, Prevotella_7, Rothia, Actinomyces, Porphyromonas, Granulicatella, Prevotella, Leptotrichia*, and *Neisseria*. Zhang's research showed that Moraxella, Streptococcus, and Actinobacteria were the major taxonomic groups in the lung microbiome of COPD patients at both the phylum and genus levels (1). The abundance of Moraxella is considered to have a significant positive correlation with the percentage of sputum neutrophils (1). High proportions of Prevotella, Veillonella, and Actinomyces species have been observed in COPD and healthy people, but the difference is not significant (3, 32); our research is consistent with this finding. The occurrence of COPD exacerbation events may be related to the reduction in microbiome diversity and increased proportion of *Proteobacteria* (33, 34).

After 7 days of SCS treatment, the bacterial abundance of *Sorangium, Acidibacter*, and *Fretibacterium* decreased at the genus level. After 14 days of SCS treatment, the bacterial abundances of *Prevotella_2, Bergeyella, Corynebacterium_1*, and *Ruminococcaceae_UCG-014* were decreased at the genus level, and an increase in the bacterial abundance of the Clostridiales_vadinBB60_group was observed at the family level. The linear discriminant analysis effect size (LEfSe) algorithm showed that after treatment for 14 days, *Sphingobacterium* increased in the SCS group, and Corynebacterium_1 (genus level), Bacillales (order level), and Lactobacillales (order level) decreased in the ICS group. *Prevotella* and *Corynebacterium* spp. are considered to be common colonizers of healthy airways (35, 36). Other bacteria that differ in relative abundance have not been found to be associated with lung infections to date. In addition, the levels of the abovementioned bacteria with different abundances were <1% in each group of samples. Therefore, we consider that the ICS and SCS will have similar effects on the relative abundance of lung bacteria.

We further analyzed the impact of the two different medication routes on microbial diversity, and the alpha diversity results showed that there were no differences in bacterial abundance between these two treatments at 7 and 14 days. Furthermore, we analyzed the effects of two different glucocorticoid usages on the beta diversity of the microbiome in patients with COPD, including PCA, PCOA, ANOSIM statistical, PERMANOVA statistical, and NMDS analyses. Our results showed that the bacterial abundance of each group was not significantly different in terms of beta diversity. Microbial diversity in the sputum of patients was decreased when acute COPD was exacerbated (1, 34), and lower values of microbial diversity of sputum with COPD were related to increasing 1-year mortality and may be used to predict the poor prognosis of hospitalized patients (14). Our results showed no significant difference between these two methods of glucocorticoid use in terms of the diversity of the microbiome in patients with COPD after 7 or 14 days of treatment. Therefore, we speculate that ICS and SCS may have similar effects on the prognosis of COPD patients. In addition, long-term use of ICS in stable COPD patients will increase the risk of pneumonia (13, 29), which may be due to lung microbiome changes (1). Our research shows that AECOPD patients' lung microbiome is similar before and after treatment with ICS; therefore, we speculate that the use of ICS in AECOPD patients may not increase the risk of pneumonia, and this speculation needs further clinical study for validation.

BugBase is a microbiome characteristics prediction tool that can make predictions based on six phenotype categories: Gram staining, oxygen tolerance, ability to form biofilms, mobile element content, pathogenicity, and oxidative stress tolerance (37). The bacterial composition predicted by BugBase demonstrated that after 14 days of treatment, the mobile genetic element (MGE) content in the sputum of SCS patients was decreased significantly compared with that in patients using ICS. MGEs are considered to be closely related to bacterial antimicrobial resistance (38, 39); thus, the relationship between ICS and resistant bacteria in COPD patients still needs further clinical research. In the treatment of AECOPD, ICS, and SCS have the same effect on aerobes, anaerobes, potentially pathogenic bacteria, bacteria related to biofilm formation, gram-negative bacteria, gram-positive bacteria, facultative anaerobes, and oxidative stress-tolerant bacteria. Our results show that ICS and SCS have the same effect on 8 phenotypic categories of bacteria; however, the results are based on BugBase predictions and need to be verified by experiments.

There are also limitations to our study. First, the sample size of this study was small, and it is necessary to increase the sample size and perform further investigation with properly designed studies. Second, the bacterial load of samples and the level of inflammatory factors in induced sputum were not detected because the induced sputum volume was insufficient. Third, there is a close relationship between COPD exacerbation and respiratory virus infection (40), and our study failed to analyze the effect of glucocorticoids on virus infection. Finally, the bronchoalveolar lavage fluid (BALF) microbiology has great significance when studying the lung microbiome (12, 41), and further investigation of the microbiome via analysis of AECOPD patients' BALF is needed.

In conclusion, by using a bioinformatics method to analyze 16S data from AECOPD patients, our study showed that there were similar effects of ICS and SCS on the sputum microbiome in patients with AECOPD, and their effects on bacterial abundance and microecological diversity were similar. Additionally, ICS has little effect on the lung microbiome of AECOPD, it may be an advantage of ICS in the treatment of AECOPD.

## Data Availability Statement

The datasets presented in this study can be found in online repositories. The names of the repository/repositories and accession number(s) can be found in the article/Supplementary Material.

## Ethics Statement

The studies involving human participants were reviewed and approved by Ethics Committee of the First Affiliated Hospital of Guangxi Medical University in 2017. The patients/participants provided their written informed consent to participate in this study.

## Author Contributions

NM was responsible for part of data analysis and article writing. YQ and XL was responsible for data collection. ML, JB, and JD reviewed and edited the manuscript. ZH designed, conducted, supervised the study, and reviewed the manuscript. All authors contributed to the article and approved the submitted version.

## Conflict of Interest

The authors declare that the research was conducted in the absence of any commercial or financial relationships that could be construed as a potential conflict of interest.
